# Detection of amoxicillin-resistant and multidrug-resistant Escherichia coli in young Australian bats (grey-headed flying fox, Pteropus poliocephalus)

**DOI:** 10.1099/mic.0.001703

**Published:** 2026-05-14

**Authors:** Fiona K. McDougall, Wayne S. J. Boardman, Michelle L. Power

**Affiliations:** 1 School of Natural Sciences, Faculty of Science and Engineering, Macquarie University, Sydney, NSW 2109, Australia; 2 School of Animal and Veterinary Sciences, Faculty of Sciences, Engineering and Technology, University of Adelaide, Roseworthy, SA 5371, Australia

**Keywords:** antimicrobial resistance, conservation, extraintestinal pathogenic *Escherichia coli*, fruit bat, One Health, wildlife rehabilitation

## Abstract

Antimicrobial-resistant *Escherichia coli* have now infiltrated the gut microbiomes of wild animals globally, posing a threat to the health and conservation of endangered species. In previous studies of an Australian fruit bat species, the grey-headed flying fox (GHFF; *Pteropus poliocephalus*), the prevalence of amoxicillin-resistant *E. coli* was significantly higher (77.4%) in GHFF pups from South Australia (SA) compared to adult GHFF (3.8%). In rehabilitation settings, amoxicillin is one of the most frequently administered antibiotics to GHFF. Our study aimed to determine the prevalence and genetic characteristics of amoxicillin-resistant *E. coli* in two additional cohorts of young GHFF: hand-reared pups from New South Wales (NSW) undergoing rehabilitation for release back to wild habitats (*n*=25) and older juveniles from SA in a pre-release facility (*n*=30), and compare these with previous findings in GHFF. Amoxicillin-resistant *E. coli* were cultured from GHFF faecal samples using amoxicillin-supplemented media. Isolates confirmed as *E. coli* by PCR underwent whole-genome sequencing and antimicrobial susceptibility testing. Amoxicillin-resistant *E. coli* were identified in 40.0% (10 out of 25) of NSW GHFF pups and 3.3% (1 out of 30) of SA juvenile GHFF. Twelve amoxicillin-resistant *E. coli* isolates were obtained from 11 bats, with all belonging to anthropogenic-associated lineages. Additional resistance to amoxicillin–clavulanic acid, cephalosporins, tetracyclines, fluoroquinolones and trimethoprim–sulfamethoxazole was detected across the 12 isolates. Overall, 33.3% of GHFF *E. coli* were multidrug-resistant, and 66.7% had extraintestinal pathogenic traits and were classified as opportunistic pathogens. These findings indicate that the prevalence of amoxicillin-resistant *E. coli* is significantly higher in GHFF pups than in juvenile and adult GHFF, and carriage may not be long-term. Further research is required to examine the prevalence and dynamics of antibiotic-resistant *E. coli* in GHFF pups as they mature. The infiltration of antibiotic-resistant *E. coli* into GHFF microbiomes presents yet another threat to this endangered species and highlights the need for good antimicrobial stewardship when treating wildlife.

## Data Summary

All whole-genome sequencing raw data have been uploaded to the NCBI SRA database under BioProject PRJNA1241462 (https://www.ncbi.nlm.nih.gov/bioproject/PRJNA1241462) and BioSamples SAMN47556985 to SAMN47556996. Assembled genomes are available at EnteroBase (http://enterobase.warwick.ac.uk/species/index/ecoli) under EnteroBase Barcodes ESC_JB6322AA to ESC_JB6327AA and ESC_JB6334AA to ESC_JB6339AA. Individual isolate EnteroBase Barcodes, NCBI SRA and assembly accession numbers are provided in Table S2, available in the online Supplementary Material.

## Introduction

The infiltration of antimicrobial-resistant *Escherichia coli* into the gut microbiomes of wild animals globally highlights the significant One Health issue of antimicrobial resistance (AMR) dissemination across humans, domestic animals, wildlife and the environment [1,2]. Increasing anthropogenic microbial pollution and AMR in the environment poses a threat to wildlife health and the conservation of endangered species [3,4]. In Australia, antimicrobial-resistant *E. coli* have been detected in multiple wild animal species, including fruit bats [grey-headed flying fox (GHFF), *Pteropus poliocephalus*] [5,6], marine mammals (Australian sea lion, *Neophoca cinerea*, and Australian fur seal, *Arctocephalus pusillus doriferus*) [7], birds (silver gulls, *Chroicocephalus novaehollandiae*) [8,9] and reptiles (green sea turtles, *Chelonia mydas*) [10]. Many of these *E. coli* isolates exhibited resistance to clinically important antibiotics such as fluoroquinolones, beta-lactams (including amoxicillin, cephalosporins and carbapenems), trimethoprim plus sulphonamides, aminoglycosides and tetracyclines [5,6, 8,11].

Phylogenetic analysis of antimicrobial-resistant *E. coli* detected in wild animals typically shows that these strains belong to anthropogenic-associated lineages, and many are strains that cause clinical infections in humans [5,6, 8, 9]. These anthropogenic-associated *E. coli* isolates are frequently characterized as extraintestinal pathogenic *E. coli* (ExPEC) strains due to the presence of specific virulence factors (VFs) that facilitate extraintestinal infections, including in the blood, urinary tract, wounds, respiratory tract and meningitis [12]. Antimicrobial-resistant ExPEC have the potential to cause infections in wild animals, as well as humans, and pose a conservation threat to endangered species [3,4]. Within the Australian context, a recent study identified closely related extraintestinal *E. coli* clusters that were shared by multiple host types, including humans, wild animals and/or companion animals [13]. Notable shared ExPEC clusters by humans and wildlife in Australia include the highly clonal and/or pathogenic *E. coli* sequence types (STs) ST131, ST457, ST963 and ST1193 [13].

The GHFF is a large tree-roosting fruit bat endemic to eastern Australia that forms very large colonies containing up to 50,000 individuals [14]. Many GHFF colonies are located in urban environments, creating opportunities for connectivity between people and GHFF, including bi-directional transmission of bacterial pathogens [5,15]. Currently, the GHFF is listed as vulnerable to extinction due to a declining population and numerous ongoing threats, including habitat loss, extreme heat events, droughts, wildfires, food shortages, electrocution on powerlines and entanglement in fruit tree netting and barbed-wire fencing [14].

In a previous study, the prevalence of *E. coli* exhibiting amoxicillin resistance was reported as 3.8% in adult GHFF from South Australia (SA) and New South Wales (NSW) [5]. A subsequent study of GHFF pups from SA that were in care (orphans undergoing rehabilitation) reported a significantly higher prevalence (77.4%) of amoxicillin-resistant *E. coli* [6]. Multidrug resistance was common in amoxicillin-resistant *E. coli* from GHFF (38.5% isolates from adults and 59.2% isolates from pups), and the majority of strains were characterized as ExPEC (69.2 and 53.1% of isolates from adults and pups, respectively) [5,6]. Whole-genome sequencing (WGS) and phylogenetic analysis showed that amoxicillin-resistant *E. coli* from GHFF typically belong to anthropogenic-associated lineages, and the majority of ExPEC strains were associated with extraintestinal infections in humans and/or domestic animals [5,6].

During the GHFF annual birthing season (October to February), hundreds or sometimes thousands of orphaned and/or injured GHFF pups come into care, where they are hand-reared and subsequently released back into wild habitats to join GHFF colonies [16]. Within these rehabilitation settings, antibiotic treatment may be required to treat bacterial infections, and amoxicillin (+/− clavulanic acid) is one of the most frequently administered antibiotics to GHFF pups [17]. Sub-optimal antimicrobial stewardship may also be occurring, with reports of prophylactic administration of amoxicillin+clavulanic acid to GHFF pups by wildlife care personnel without specific veterinary prescribing [6]. In GHFF colonized by antimicrobial-resistant *E. coli*, antibiotic administration may have the unintentional side effect of selecting for resistant ExPEC and allowing these opportunistic pathogens to establish infections [18,19].

This study aimed to determine the prevalence and genetic characteristics of amoxicillin-resistant *E. coli* in two additional cohorts of GHFF – pups from NSW and older juveniles from SA – and contrast carriage with that previously reported in GHFF pups from SA and adult GHFF from SA and NSW. The study will address knowledge gaps around how widespread the high prevalence of antibiotic-resistant *E. coli* is across different cohorts of young GHFF and if prevalence decreases when pups mature. The study findings will further inform GHFF rehabilitation management practice, wildlife rehabilitation policies and antimicrobial stewardship guidelines to improve rehabilitation and treatment outcomes for GHFF.

## Methods

### GHFF faecal sampling

Bat faecal samples (*n*=56) were collected from young orphaned and/or injured GHFF that had been rescued and were being rehabilitated for release back into wild bat colonies. Bats were sampled at two locations: NSW (*n*=26 faecal samples) between 15 October 2018 and 3 April 2019, and SA (*n*=30 faecal samples) on 19 April 2019 (Table S1). The NSW bats were rescued from multiple locations across NSW and entered into care with eight different carers, whereas the SA bats were all rescued in Adelaide, SA, and were co-housed in a large pre-release cage (Table S1). Faecal samples were collected using the FecalSwab^™^ system (COPAN, Brescia, Italy) – 1 NSW bat was sampled twice (7 days apart), and the remaining bats (*n*=54) were sampled once, resulting in a total of 56 faecal samples from 55 bats (Table S1). The GHFF faecal samples from NSW were collected from clean paper or towels placed underneath bats after they entered into wildlife care, and GHFFs in SA were sampled by rectal swab, just prior to release back into a wild bat colony. Aliquots of bat faecal swab media were stored in 35% glycerol and frozen at −80 °C until selective culture was performed. Data collected for GHFF individuals included medical history and date of entry to care (NSW GHFF only), animal weights and forearm length (FAL) measurements (all bats) and bat age, which was estimated using FAL with the Pinson and Kerr ageing chart [20] (Table S1).

### Selective culture for amoxicillin-resistant enteric bacteria

GHFF faecal samples were cultured using media supplemented with amoxicillin to detect amoxicillin-resistant *E. coli.* Faecal swab media plus 35% glycerol (0.4 ml) was added to 10 ml Luria–Bertani broth (Difco Laboratories, Detroit, MI, USA) supplemented with 10 mg l^−1^ amoxicillin (Sigma, St. Louis, MO, USA) and subsequently onto Chromocult Coliform Agar (Merck Millipore, Burlington, MA, USA) supplemented with 10 mg l^−1^ amoxicillin (Sigma, St. Louis, MO, USA), as previously described [6]. Genomic DNA was extracted from *E. coli* isolates using the ISOLATE II Genomic DNA Kit (Bioline, London, UK), as previously described [6]. Isolates were confirmed as *E. coli* and assigned to a phylogroup (A, B1, B2, C, D, E, F or cryptic clade I) using the Clermont quadruplex phylotyping PCR method [21], as previously described [6].

### WGS of amoxicillin-resistant isolates

Genomic DNA concentrations were measured using a Qubit dsDNA HS Assay kit (Invitrogen, Waltham, MA, USA). WGS was performed using the Illumina NextSeq 1000 system at the Ramaciotti Centre for Genomics (Sydney, NSW, Australia). Velvet short-read assembler version 1.2 [22] was used to assemble raw sequence reads as *de novo* genome sequences in Geneious Prime software version 2024 (Biomatters Limited, Auckland, New Zealand). Raw sequence reads for all *E. coli* isolates were uploaded to the NCBI Sequence Read Archive (SRA) under BioProject PRJNA1241462 and BioSample IDs SAMN47556985–SAMN47556996 (available at https://www.ncbi.nlm.nih.gov/bioproject/PRJNA1241462). Isolate raw sequence reads were also uploaded to EnteroBase (EnteroBase Barcodes ESC_JB6322AA to ESC_JB6327AA and ESC_JB6334AA to ESC_JB6339AA) (http://enterobase.warwick.ac.uk/species/index/ecoli). Individual isolate EnteroBase Barcodes, NCBI SRA and NCBI assembly accession numbers are provided in Table S2.

### Bacterial strain characterization and detection of antibiotic resistance genes

The bat *E. coli* isolates were assigned an ST [Achtman 7-gene multilocus sequence typing (MLST)], phylogroup (ClermonTyping), serotype (O antigen:H antigen) and FimH type in EnteroBase (all available at https://enterobase.warwick.ac.uk/species/index/ecoli) [23,25]. Plasmids were identified in *E. coli* isolates using PlasmidFinder Version 2.1 (https://cge.food.dtu.dk/services/PlasmidFinder/) [26,27].

The *E. coli* isolate genome assemblies were screened for VFs using VirulenceFinder 2.0 (https://cge.food.dtu.dk/services/VirulenceFinder/) [28] and ABRicate VFDB – Galaxy Version 1.0.1 (available at https://usegalaxy.org.au) [29]. To identify isolates with ExPEC characteristics, each isolate assembly was assessed for the presence of 27 ExPEC-associated VFs: iron acquisition (*fyuA/irp/ybt*, *ireA*, *iroN*, *iutA/iucA* and *sitA*), adhesins (*afa/dra*, *fimH*, *iha*, *papA/papC*, *sfa/foc *and *tsh*), invasins (*gimB* and *ibeA*), toxins (*astA*, *clb*, *cnf1*, *hly*, *sat*, *usp* and *vat*), capsule (*kpsM II*), protectins (*iss*, *neuC* and *traT*) and miscellaneous (*ompT*, *pic* and *malX*) [12,30, 31]. Isolate assemblies were also assessed for additional VFs that may be present in some ExPEC subtypes (bacteriocins, colicins, microcins, *chuA*, *clpK1/clpK2*, *lpfA* and *senB*). Isolates were also screened for the presence of intestinal pathogenic *E. coli*-associated VFs (including *aggR*, *air*, *bfpA*, *eae*, *eilA*, *shuA/shuT* and *stx*).

Isolates were classified as an ExPEC pathotype if they carried two or more of the five key ExPEC VFs (*afa/dra*, *iutA*, *kpsM II*, *papA/papC* and *sfa/fo*c) [30]. Isolates carrying fewer than 2 key ExPEC VFs were classified as follows: ‘ExPEC-potential’ if they carried 5 or more of the 27 ExPEC-associated VFs, ‘ExPEC-low potential’ if they carried fewer than 5 ExPEC-associated VFs but 5 or more total VFs (including additional VFs) or ‘Low pathogenicity’ if they carried fewer than 5 total VFs.

All isolates were screened for antimicrobial resistance genes (ARGs) using ResFinder Version 4.7.2 (available at https://genepi.food.dtu.dk/resfinder) [27,32], and ARGs in *E. coli* isolates were also identified in EnteroBase using the AMR analysis tool (available at https://enterobase.warwick.ac.uk/species/index/ecoli) [23].

### Antimicrobial susceptibility testing

The amoxicillin-resistant *E. coli* isolates underwent antimicrobial susceptibility testing (AST) according to the disc diffusion method described by the European Committee on Antimicrobial Susceptibility Testing (EUCAST) [33], as previously described [5]. Isolates were screened against a panel of 16 antimicrobial susceptibility discs (Oxoid, Basingstoke, UK): ampicillin (AMP10), amoxicillin+clavulanic acid (AMC30), cephalexin (CL30), cephazolin (KZ30), cefotaxime (CTX5), imipenem (IPM10), amikacin (AK30), gentamicin (CN10), streptomycin (S25), spectinomycin (SH25), trimethoprim (W5), trimethoprim+sulfamethoxazole (SXT30), chloramphenicol (C30), tetracycline (TE30), nalidixic acid (NA30) and ciprofloxacin (CIP5). Multidrug resistance was defined as acquired phenotypic resistance to one or more antibiotics in at least three antimicrobial categories [34].

### Phylogenetic analysis

For each GHFF *E. coli* isolate, closely related isolates were identified in EnteroBase by searching for isolates with the same ST using the Achtman 7-gene MLST scheme (http://enterobase.warwick.ac.uk/species/ecoli/search_strains?query=st_search) [23]. Phylogenetic analysis was performed using GrapeTree to construct a rapid neighbour-joining (RapidNJ) minimum spanning tree based on the core genome multilocus sequence typing (cgMLST) V1+Hierarchical Clustering (HierCC V1) scheme from EnteroBase (available at https://enterobase.warwick.ac.uk/species/index/ecoli) [35].

### Statistical analysis

All data analysis and descriptive statistics for GHFF data were performed using Microsoft Excel (Microsoft, Redmond, USA). To test for statistical differences in the frequency of amoxicillin-resistant *E. coli* in the two cohorts of young GHFF (NSW pups and SA juveniles), Fisher’s exact test was used in R (4.5.1) (https://CRAN.R-project.org/package=epiR) and RStudio (2025.05.1+513) (https://posit.co/download/rstudio-desktop/), with *P*<0.05 being considered statistically significant.

## Results

### GHFF characteristics

**Fig. 1.**
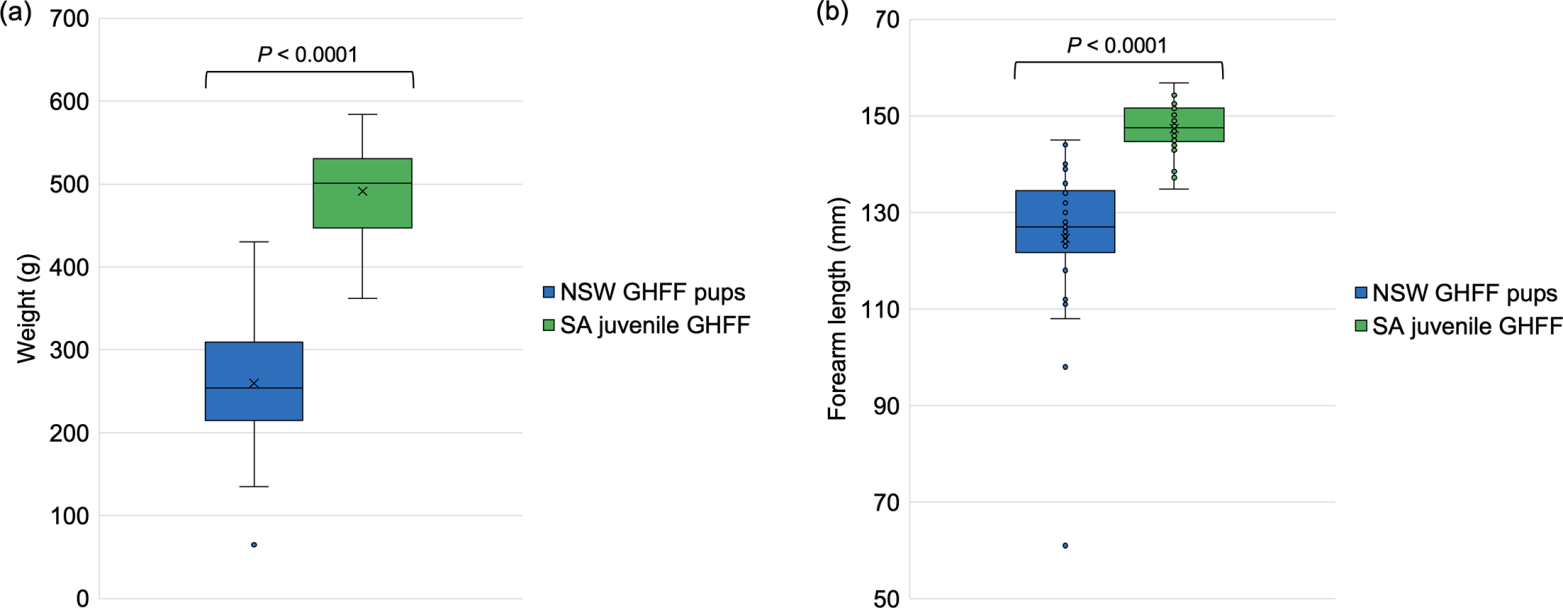
Box and whisker plots displaying the body weights (g) and FAL (mm) for two additional cohorts of young bats (GHFF; *P. poliocephalus*): GHFF pups in care in NSW (*n*=25) and juvenile GHFF in pre-release facilities in SA (*n*=30). SA juvenile GHFF (*n*=30) had significantly higher body weights (t-test, *P*<0.0001) than NSW GHFF pups (*n*=25). Similarly, SA juvenile GHFF (*n*=30) had significantly longer FALs (t-test, *P*<0.0001) than NSW GHFF pups (*n*=25). Boxes indicate the interquartile ranges. X indicates the mean weight or FAL. (a) Body weight (g). (b) FAL (mm).

GHFF pups in care with bat carers in NSW were sampled between 0 and 59 days after entry to care, with the majority (16 of 25, 60.0%) being pre-weaned (aged <12 weeks) (Table S1). GHFFs in SA were an older, weaned cohort that were sampled after transition to a pre-release facility (from previously being in care with bat carers) in preparation for release back into the wild bat colony in Adelaide (SA) (Table S1). The mean estimated age for NSW GHFF pups at the time of sampling was 78 days (range 4–127 days); however, as the majority (19 out of 30) of the SA GHFF were estimated to be >140 days old (the limit of the Pinson and Kerr ageing chart), a mean estimated age could not be calculated (range 92–140+ days) (Table S1). The mean GHFF body weights correlated with the age difference of the two cohorts, with GHFF in NSW (*n*=25) having a mean weight of 259.8 g, whereas the GHFFs in SA (*n*=30) were significantly heavier (t-test, *P*<0.0001), with a mean weight of 491.3 g (Fig. 1 and Table S1). Similarly, the mean FAL of NSW GHFF (*n*=25) was 124.6 mm, whereas the SA GHFFs (*n*=30) were significantly larger (t-test, *P*<0.0001), with a mean FAL of 147.0 mm (Fig. 1 and Table S1). For simplicity, the predominantly younger group of NSW GHFF bats in care will be referred to as ‘NSW GHFF pups’, and the predominantly older pre-release group of GHFF from SA will be referred to as ‘SA juvenile GHFF’ from hereon.

### Amoxicillin-resistant *E. coli* detected in bats

Twelve amoxicillin-resistant *E. coli* isolates were obtained from the 55 young GHFF, with 11 detected in 10 pups (NSW GHFF) – 1 pup carried 2 different *E. coli* strains (10 out of 25, 40.0%) – and 1 isolate was detected in an SA juvenile GHFF (1 out of 30, 3.3%). The frequency of amoxicillin-resistant *E. coli* was significantly higher (Fisher’s exact test, *P*=0.001) in NSW GHFF pups (40.0%) compared to SA juvenile GHFF (3.3%). For the NSW GHFF pups, 6 amoxicillin-resistant *E. coli* isolates were obtained from 15 faecal samples collected at entry to care (≤2 days in care – 6 out of 15, 40.0%), and 4 amoxicillin-resistant *E. coli* isolates were obtained from 11 faecal samples collected during care (≥8 days in care – 4 out of 11, 36.4%) (Table S1). The NSW GHFF pup that was sampled twice (at entry to care and 7 days after entry) carried an amoxicillin-resistant *E. coli* strain at the first sampling but not at the second sampling. The mean estimated age of NSW GHFF pups with amoxicillin-resistant *E. coli*-positive faecal samples was 75.5 days (range 36–105 days), and similarly, the mean estimated age of NSW GHFF pups negative for amoxicillin-resistant *E. coli* was 74.9 days (range 4–127 days) (Table S1). For NSW GHFF pups, amoxicillin-resistant *E. coli* were detected in faecal samples from carers located in the city of Sydney, NSW (8 of 20, 40.0%), and carers located in regional NSW (2 of 6, 33.3%) (Table S1). For NSW carers with only one pup in care, 33.3% (2 of 6) faecal samples were positive for amoxicillin-resistant *E. coli*, and similarly, 40.0% (8 of 20) positive samples were from NSW carers with two or more pups in care at the same time (Table S1). Only 1 of the 55 sampled bats received systemic antibiotics – amoxicillin+clavulanic acid for treatment of pneumonia – and this individual did not carry amoxicillin-resistant *E. coli* (Table S1).

The 12 amoxicillin-resistant *E. coli* isolates underwent WGS, genomic characterization and AST (Table 1). The 12 isolates belonged to 11 different STs and 4 phylogroups, with the predominant being phylogroup A (*n*=6), followed by phylogroup B2 (*n*=4), phylogroup B1 (*n*=1) and phylogroup D (*n*=1) (Table 1). Analysis of virulence genes determined that 1 of 12 isolates was classified as ExPEC (ST636 O83:H7 carried 2 key ExPEC VFs), 7 isolates were deemed potential ExPEC and 4 were deemed to have low pathogenicity (Table 1). Notable ExPEC-associated VFs included the following: iron acquisition – yersiniabactin (4 out of 12, 33.3%), adhesin – *papC* (4 out of 12, 33.3%), toxin – *vat* (4 out of 12, 33.3%), invasin – *ibeA* (2 out of 12, 16.7%) and capsule – *kpsMII* (1 out of 12, 8.3%) (Table 1).

**Table 1.** Genomic characterization of 11 amoxicillin-resistant *E. coli* isolates from GHFF faecal samples. Phenotypic AMR was determined according to the EUCAST disc diffusion method. Antibiotics: AMP, ampicillin; AMX, amoxicillin; AMC, amoxicillin plus clavulanic acid; CL, cephalexin; KZ, cephazolin; CTX, cefotaxime; SH, spectinomycin; SXT, trimethoprim plus sulfamethoxazole; W, trimethoprim; TE, tetracycline; Int, intermediate resistance. Key ExPEC-associated VFs are in bold font. PG, phylogroup.

Isolate ID	PG	ST	Serotype (O:H)	FimH type	Acquired ARGs	Phenotypic AMR	VFs	Pathotype (no. of ExPEC VFs/no. of other VFs)
FF1509A1	B2	636	O83:H7	fimH75	*bla*_TEM-40_	AMP, AMX, AMC	*chuA*, *fimH*, *fyuA/irp/ybt*, *ibeA*, *kpsD/kpsE/**kpsMII_K5***, *ompT*, ***papC***, *sitA*, *usp*, *vat*	ExPEC (9/1)
FF1527A	A	6113	O159:H23	fimH86	*bla*_TEM-1B_, *tet(A)*	AMP, AMX, AMC, TE	*cia*, *cma*, *cvaC*, *fimH*, *hlyE/hlyF*, *iroN*, *iss*, *lfpA*, *mchF*, *ompT*, *sitA*, *traT*	ExPEC-potential (7/5)
FF1546A1	B2	491	O103:H45	fimH5	*bla*_TEM-40_	AMP, AMX, AMC	*chuA*, *fimH*, *fyuA/irp/ybt*, *iss*, *ompT*, ***papC***, *pic*, *sitA*, *vat*	ExPEC-potential (8/1)
FF1548A	B2	491	O103:H45	fimH5	*bla*_TEM-40_	AMP, AMX, AMC	*chuA*, *fimH*, *fyuA/irp/ybt*, *iss*, *ompT*, ***papC***, *pic*, *sitA*, *vat*	ExPEC-potential (8/1)
FF1556A	B1	155	O58:H5	fimH121	*bla*_TEM-40_	AMP, AMX, AMC	*fimH*, *hlyE*, *lpfA*	Low pathogenicity (2/1)
FF1557	A	5625	ONT:H10	fimH38	*aadA1*, *bla*_TEM-135_, *dfrA15*, *qnrS13*, *sul3*, *tet(A)*	AMP, AMX, W, SXT, CIP(Int), TE, SH (Int)	*fimH*, *hlyE*, *lpfA*	Low pathogenicity (2/1)
FF1560	D	10182	ONT:H48	fimH49	*bla*_TEM-40_	AMP, AMX, AMC	*chuA*, *air*, *eilA*, *fimH*, *hlyE*, *iss*, *ompT*, *shuA/shuT/shuX*, *traT*	ExPEC-potential (5/4)
FF1603B	B2	706	O51:H1	fimH229	*bla*_TEM-40_	AMP, AMX, AMC	*chuA*, *clbB*, *fimH*, *fyuA/irp/ybt*, *ibeA*, *iss*, *ompT*, ***papC***, *sitA*, *usp*, *vat*	ExPEC-potential (9/2)
FF1607A	A	10	ONT:H12	fimH24	*bla*_CMY-2_	AMP, AMX, AMC, CL, KZ, CTX	*cia*, *fimH*, *hlyE*, *iss*, *sitA*	ExPEC-low potential (4/1)
FF1608A	A	15381	O15:H11	fimH41	*bla*_TEM-1B_, *tet(A)*	AMP, AMX, TE	*fimH*	Low pathogenicity (1/0)
FF1608B	A	1316	O21:H55	Not found	*bla*_TEM-40_, *dfrA14*, *qnrS1*	AMP, AMX, AMC, CIP(Int), W	*clpK1/clpK2*, *hlyE*, *sitA*	Low pathogenicity (2/1)
FF1941	A	10	O113:H4	fimH54	*bla*_TEM-1B_	AMP, AMX	*fimH*, *hlyE*, *iss*, *ompT*, *sitA*	ExPEC-potential (5/0)

All 12 isolates carried acquired beta-lactamase genes, which conferred phenotypic resistance to ampicillin and amoxicillin, and resistance to amoxicillin plus clavulanic acid for 9 of 12 isolates (75.0%), as determined by the EUCAST disc diffusion AST method (Table 1). One isolate (FF1607A, ST10) carried *bla*_CMY-2_, which also conferred resistance to first- and third-generation cephalosporins (Table 1). Additional acquired genes conferred resistance to tetracycline (3 out of 12, 25.0%), ciprofloxacin (2 out of 12, 16.7%), trimethoprim+/−sulfamethoxazole (2 out of 12, 16.7%) and spectinomycin (1 out of 12, 8.3%), and multidrug resistance was observed in 3 isolates (4 out of 12, 33.3%) (Table 1). No isolates exhibited phenotypic resistance to carbapenems, chloramphenicol or gentamicin.

### Phylogenetic analysis of amoxicillin-resistant *E. coli* isolates with ExPEC characteristics

**Fig. 2.**
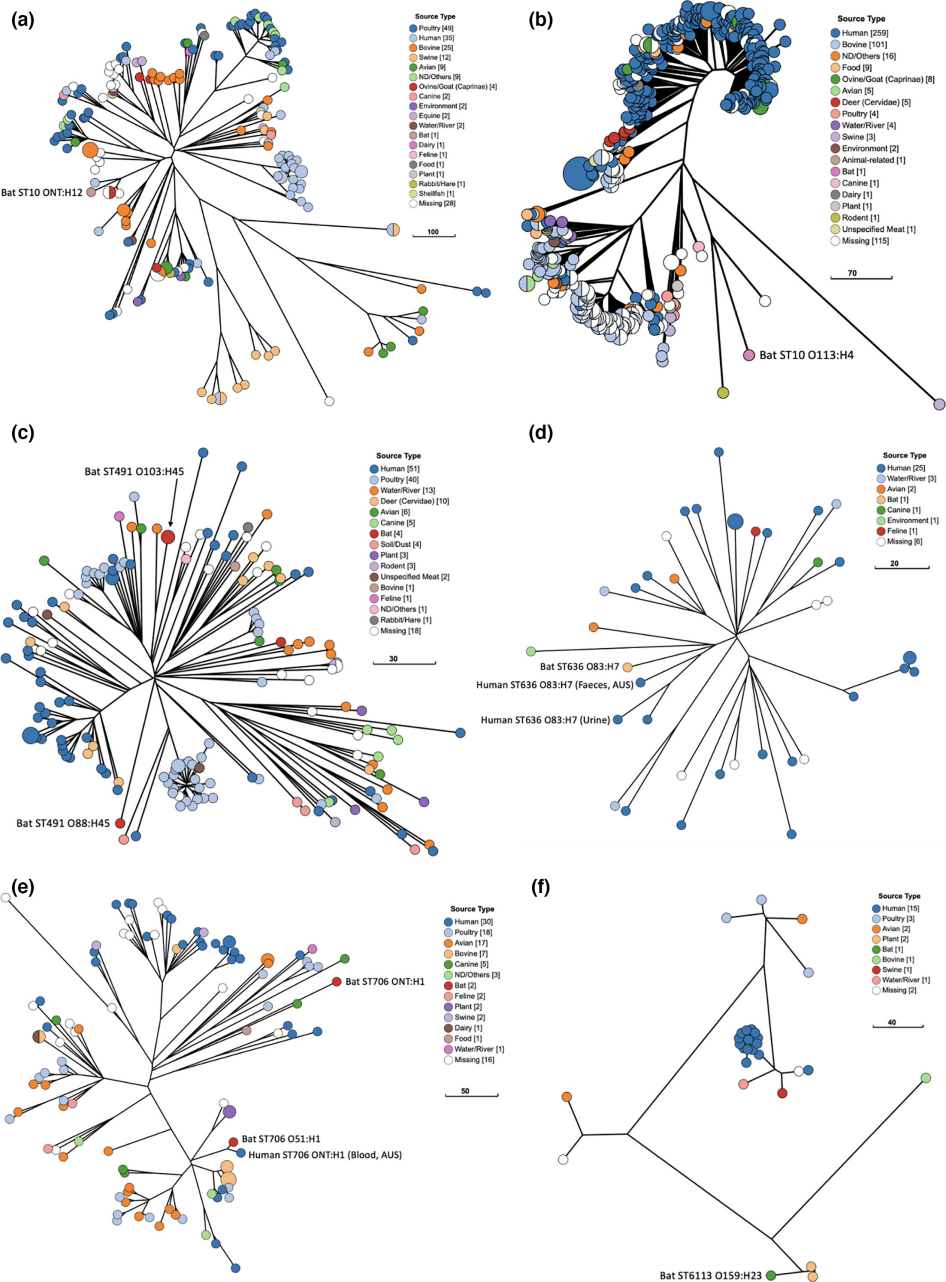
Phylogenetic analysis of amoxicillin-resistant ExPEC isolates from young GHFFs and closely related isolates identified in EnteroBase. All phylogenies were constructed with GrapeTree using a RapidNJ minimum spanning tree based on the cgMLST V1+Hierarchical Clustering (HierCC V1) scheme in EnteroBase (available at https://enterobase.warwick.ac.uk/species/index/ecoli). (a) Bat isolate ST10 ONT:H12 and related isolates (*n*=187). (b) Bat isolate ST10 O113:H4 and related isolates (*n*=538). (c) Bat isolate ST491 (*n*=2) and related isolates (*n*=163). (d) Bat isolate ST636 O83:H7 and related isolates (*n*=47). (e) Bat isolate ST706 and related isolates (*n*=107). (f) Bat isolate ST6113 O159:H23 and closely related isolates (*n*=28).

Of the two ST10 bat isolates, ST10 ONT:H12 clustered with four other ST10 ONT:H12 isolates, including three sourced from human faeces (*n*=1), livestock (*n*=1) and animal manure (*n*=1) (EnteroBase Barcodes ESC_TB4262AA, ESC_UA2154AA and ESC_HB3199AA, respectively) (Fig. 2a). ST10 O113:H4 was most closely related to three isolates, all also ST10 O113:H4, one sourced from a wild rat, one from an unknown source and one from swine (EnteroBase Barcodes ESC_IB6171AA, ESC_KA5443AA and ESC_ZA0221AA, respectively) (Fig. 2b).

Both ST491 O103:H45 bat isolates were determined to be the same strain and exhibited identical genomic characteristics and phenotypic resistance profiles (Table 1). The two ST491 isolates were sourced from two co-housed bat pups in care with the same carer for 25 days, indicating that either both pups acquired the ST491 strain from the same source or that bat-to-bat transmission has occurred (Table S1). These isolates were most closely related to an ST491 O103:H45 isolate sourced from river water in Japan (EnteroBase Barcode ESC_SA6786AA) and an isolate sourced from human urine (EnteroBase Barcode ESC_BB6938AA) (Fig. 2c). The GHFF ST491 O103:H45 isolates were only distantly related to another ST491 isolate (O88:H45) previously sourced from an Australian GHFF in 2015 (EnteroBase Barcode ESC_WA3810AA) (Fig. 2c).

The bat ST636 ExPEC isolate was clustered with five other ST636 O83:H7 isolates, three of which originated from human sources (urine, *n*=1, and faeces, *n*=2), one from a wild bird and one from an environmental source (Fig. 2d). The most closely related isolate (EnteroBase Barcode ESC_ZA6265AA) was also detected in Australia (human faeces) and had an almost identical VF profile (lacked *papC*), but it did carry a different resistance profile (*bla*_CTX-M-3_) than the bat ST636 isolate (*bla*_TEM-40_).

Bat isolate ST706 O51:H1 was very closely related to one isolate (ST706 ONT:H1) sourced from a human bloodstream infection in Australia (EnteroBase Barcode ESC_UA5632AA) (Fig. 2e). The two ST706 isolates shared almost identical VF profiles, including yersiniabactin, *ibeA*, *papC*, *usp* and *vat*; however, the human ST706 isolate carried a different resistance gene profile. These two ST706 isolates were only distantly related to a previously sourced Australian bat ST706 isolate (ONT:H1) (EnteroBase Barcode ESC_WA3811AA) (Fig. 2e).

The bat ST6113 O159:H23 isolate was most closely related to two isolates sourced from vegetable crops (EnteroBase Barcodes ESC_JA2092AA and ESC_RB9643AA) and a bovine-sourced isolate (EnteroBase Barcode ESC_DB3843AA), rather than with the multiple human- and poultry-sourced ST6113 isolates (Fig. 2f). The three vegetable- and bovine-sourced ST6133 isolates lacked numerous VFs and resistance genes that were present in the bat ST6133 isolate, including *cia*, *cma*, *cvaC*, *iroN*, *iss*, *mchF*, *ompT*, *sitA*, *traT*, *bla*_TEM-1B_ and *tet(A)*. PlasmidFinder and BLASTn searches determined that the bat ST6113 isolate has acquired multiple plasmids, including IncFIB, IncFII and an avian pathogenic *E. coli*-associated colicin plasmid (GenBank accessions CP087565 and CP123267).

In addition to the bat ST10182 bat isolate, only four other ST10182 isolates (all ONT:H48) were identified in EnteroBase, which included two sourced from red foxes in Sweden (EnteroBase Barcodes ESC_QB5849AA and ESC_QB5857AA) and two from livestock in the UK (EnteroBase Barcodes ESC_DB4390AA and ESC_OA2182AA).

### Phylogenetic analysis of amoxicillin-resistant low-pathogenicity isolates

**Fig. 3.**
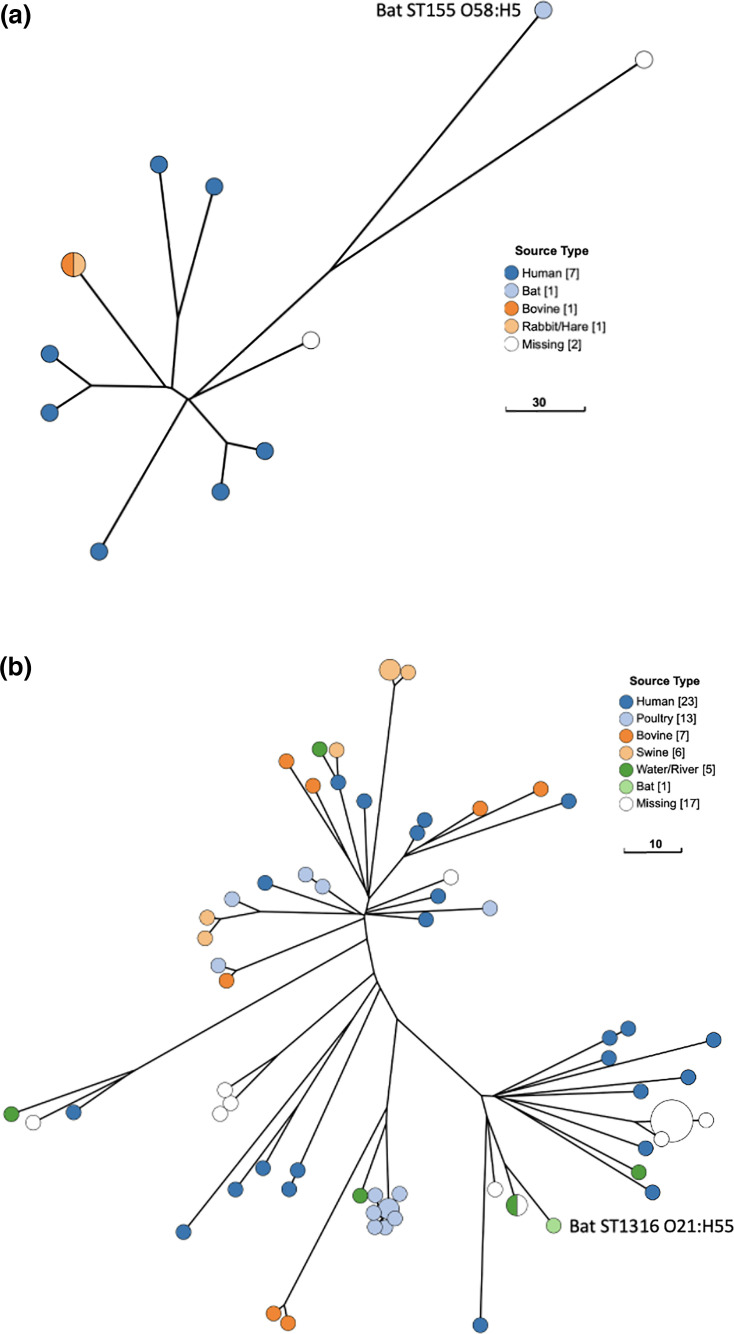
Phylogenetic analysis of amoxicillin-resistant low-pathogenicity *E. coli* isolates from young GHFFs and closely related isolates identified in EnteroBase. All phylogenies were constructed with GrapeTree using a RapidNJ minimum spanning tree based on the cgMLST V1+Hierarchical Clustering (HierCC V1) scheme in EnteroBase (available at https://enterobase.warwick.ac.uk/species/index/ecoli). (a) Bat isolate ST155 O58:H5 and related isolates (*n*=12). (b) Bat isolate ST1316 O21:H55 and related isolates (*n*=72).

The bat ST155 O58:H5 isolate was closely related to seven human-sourced isolates, including one sourced from urine (EnteroBase Barcode ESC_TA8009AA) (Fig. 3a). The ST1316 O21:H55 isolate was very closely related to four isolates identified in EnteroBase, which included an Australian septic tank-sourced isolate and a human-sourced isolate (EnteroBase Barcodes ESC_UA0086AA and ESC_XA4573AA, respectively) (Fig. 3b). Notably, none of the closely related ST1316 isolates carried any acquired resistance genes, unlike the bat ST1316 isolate which carried *bla*_TEM-40_, *dfrA14* and *qnrS1*. Insufficient isolate metadata was available in EnteroBase to determine the origins of isolates most closely related to the remaining two low-pathogenicity bat *E. coli* isolates (ST5625 ONT:H10 and ST15381 O15:H11); however, both isolates belonged to anthropogenic-associated *E. coli* lineages (Table S3).

## Discussion

This study confirms that amoxicillin-resistant *E. coli* is commonly present in the faecal microbiome of GHFF pups. However, the prevalence in the NSW GHFF pups tested in this study was lower (40.0%) than previously reported in GHFF pups from SA (77.4%) [6]. The detection of a single amoxicillin-resistant *E. coli* isolate in a juvenile GHFF from SA, with a prevalence of 3.3%, is comparable to the prevalence previously observed in adult GHFF, 3.8% [5]. These findings showed that amoxicillin-resistant *E. coli* carriage in GHFF pups is significantly higher than in juvenile and adult GHFF and suggests that carriage may not be long-term as the frequency appears to decline as the bat pups mature. However, climate events such as extreme heat events and low rainfall during the 2018–2019 pup season were shown to be associated with an increase in prevalence and diversity of amoxicillin-resistant *E. coli* in GHFF pups from SA [6]. While seasonality was not explored in this study, the SA juvenile GHFFs were from the 2020–2021 pup season, which had above-average rainfall and cool temperatures over summer, in comparison to the hot and dry conditions in the 2018–2019 summer [36]. Further research is required to examine the prevalence and dynamics of antibiotic-resistant *E. coli* in GHFF pups as they mature and transition through rehabilitation stages from entry into care and up until juveniles are released back into wild GHFF colonies, and to investigate if climate variables influence *E. coli* dynamics in young GHFF.

The amoxicillin-resistant *E. coli* isolates from young GHFF in this study had acquired beta-lactamase genes (predominantly *bla*_TEM_, 91.7%), which is comparable to previous studies [5,6]. In this study, the number of amoxicillin-resistant *E. coli* that were classified as multidrug-resistant (33.3%) was lower than in previous studies, which reported that 59.2% (SA GHFF pups) and 38.5% (adult GHFF) of amoxicillin-resistant *E. coli* were multidrug-resistant [5,6]. Although multidrug-resistant *E. coli* prevalence was lower in this study than previously reported, resistance to fluoroquinolones and third-generation cephalosporins was identified – two antibiotics classified as highest priority critically important antimicrobials by the World Health Organization [37].

The majority of amoxicillin-resistant *E. coli* isolates harboured ExPEC virulence characteristics and are considered to be opportunistic pathogens. In this study, the proportion of amoxicillin-resistant *E. coli* with ExPEC characteristics (66.7%) was comparable to previous reports in GHFF pups from SA (53.1%) [6] and adult GHFF (69.2%) [5]. Two GHFF isolates of particular concern were ExPEC ST636 O83:H7 and ST706 O51:H1, which were closely related to isolates cultured from human extraintestinal infections. Both ST636 and ST706 are reported to be associated with urinary tract infections [38,39], and ST636 with bacteraemia/sepsis in humans [40].

Phylogenetic analysis confirmed that all GHFF amoxicillin-resistant *E. coli* isolates identified in this study belonged to anthropogenic-associated lineages, indicating that the direction of transmission is from anthropogenic sources to bats [1,2]. Additionally, GHFF pups from NSW that were sampled at entry to care showed a high prevalence of amoxicillin-resistant *E. coli* (40.0%), suggesting that acquisition occurred prior to entering rehabilitation settings. These findings are in agreement with the previous study in GHFF pups from SA, which also determined that GHFF amoxicillin-resistant *E. coli* belonged to anthropogenic-associated lineages, and pups also showed high prevalence of amoxicillin-resistant *E. coli* (60.0%) at entry to care [5,6]. The acquisition of anthropogenic-associated amoxicillin-resistant *E. coli* by GHFF pups prior to entering care indicates that the most likely acquisition source is exposure to anthropogenic microbial pollution in the environment [5,6].

The detection of amoxicillin+/–clavulanic acid-resistant ExPEC in GHFF pups from NSW identifies extraintestinal infection risks but also demonstrates that the infiltration of these pathogens into GHFF pup gut microbiomes is widespread and not restricted to GHFF pups from SA [6]. This infiltration of anthropogenic-associated antibiotic-resistant ExPEC into the gut microbiomes of young GHFF is yet another threat to this endangered species [14], which may be exacerbated by other anthropogenic-driven threats, including heat waves, food shortages, drought, habitat loss and urbanization [41,42]. This study also highlights the need for good antimicrobial stewardship and judicious antibiotic administration when treating wildlife [43]. Antibiotic administration may enhance the transmission of ARGs between bacterial strains, resulting in the emergence of new antibiotic-resistant bacterial pathogens [44,45]. There is an urgent need for further research on the ecology of anthropogenic AMR pollution in wildlife and the impact of this pollution on wildlife health and conservation.

## Supplementary material

10.1099/mic.0.001703 Supplementary Material 1.

## Abbreviations

AMR: antimicrobial resistance
ARG: antimicrobial resistance gene
AST: antimicrobial susceptibility testing
cgMLST: core genome multilocus sequence typing
EUCAST: European Committee on Antimicrobial Susceptibility Testing
ExPEC: extraintestinal pathogenic *Escherichia coli*
FAL: forearm length
GHFF: grey-headed flying fox
MLST: multilocus sequence typing
NSW: New South Wales
PG: phylogroup
RapidNJ: rapid neighbour-joining
SA: South Australia
SRA: Sequence Read Archive
ST: sequence type
VFs: virulence factors
WGS: whole-genome sequencing
